# Supplementary material for: Content-rich biological network constructed by mining PubMed abstracts
Source: BMC Bioinformatics. 2004 Oct 8;5:147. doi: 10.1186/1471-2105-5-147 (PMC528731; doi:10.1186/1471-2105-5-147)
Supplement: Additional File 5 — The original Chilibot query results of the term "long-term potentiation (LTP)" and 22 other terms, limiting the latest references analyzed to the years 1990, 1995, 2000, and 2004. [file 1471-2105-5-147-S5.bz2 › chilibotAdditionalFile5/ltp2000/html/PLC_ATF.html]

 


 **PLC** and **ATF** 
  
Found 1 abstracts in PubMed,  **1 abstracts were retrieved and analyzed**.  


---

 Search Google  |
 PDF files only 
|  EDU domain only 

---

**Interactive relationship** (e.g. stimulation, inhibition, etc)

**Parallel relationship** (e.g. studied together, co-existance, homology, etc.)

- Cell migration and invasion were enhanced by uPA or  **ATF**  in a dose dependent manner, while PI  **PLC**  treatment abolished the uPA effect and inhibited migration and invasion.  Ref: 10066093 J Neurooncol, 1998
- Migration on Transwell membranes and invasion of Matrigel was then tested by pre incubating the cells with increasing concentrations of either uPA, the proteolytically inactive amino terminal fragment  **ATF**  of uPA, or the uPAR cleaving enzyme, phosphatidylinositol specific phospholipase C PI  **PLC** .  Ref: 10066093 J Neurooncol, 1998
